# Bacterial communities and signatures in the stomach and intestine of juvenile *Penaeus* (*litopenaeus*) *vannamei* shrimp affected by acute hepatopancreatic necrosis disease

**DOI:** 10.1016/j.heliyon.2024.e33034

**Published:** 2024-06-15

**Authors:** Guillermo Reyes, Betsy Andrade, Irma Betancourt, Fanny Panchana, Cristhian Preciado, Bonny Bayot

**Affiliations:** a Centro Nacional de Acuicultura e Investigaciones Marinas, CENAIM, Escuela Superior Politécnica Del Litoral, ESPOL, Guayaquil, Ecuador; b Facultad de Ingeniería Marítima y Ciencias Del Mar, FIMCM, Escuela Superior Politécnica Del Litoral ESPOL, Guayaquil, Ecuador

**Keywords:** Microbiome, Vibriosis, Shrimp probiotics, Farmed shrimp, Gastrointestinal microbiota, 16S rDNA amplicon, Biomarkers

## Abstract

Acute hepatopancreatic necrosis (AHPND) is a severe bacterial disease affecting farmed shrimp. Although various pathogenic bacteria associated with AHPND-affected shrimp have been described, little is known about the bacterial signatures in the stomachs and intestines when the disease occurs naturally. In this study, we characterized the microbiome of *P. vannamei* by high-throughput sequencing (HTS). Shrimp samples were collected from a commercial farm and divided into two groups: healthy and affected by AHPND, confirmed by PCR. Stomach and intestine samples were subjected to microbiome analysis targeting the V3–V4 region of the *16S rRNA* gene. PERMANOVA analysis revealed a significant disparity in the bacterial diversity between the stomach and intestine microbiomes of these two health conditions. Our results suggest that the significant abundance of *Vibrio brasiliensis* and *V. sinaloensis* in the intestines of affected shrimp plays a role in AHPND infection. This imbalance could be mitigated by the presence of *Pseudoalteromonas*, *Gilvimarinus,* and other members of the phylum Pseudomonadota such as Cellvibrionaceae, Psychromonadaceae, and Halieaceae, which showed significant abundance in healthy intestines. This study highlights the significance of the microbial community in the differentiation of specific microbial signatures in different organs of *P. vannamei*. These findings offer a deeper understanding of the intricate dynamics within the shrimp microbiome under these conditions, enriching our view of AHPND progression and paving the way toward future identification of probiotics tailored for more efficient management of this disease.

## 1 Introduction

The gastrointestinal microbiota of the shrimp is a complex micro-ecosystem involved in multiple fundamental biological processes of the host, including nutrition and immune system function [[1], [2], [3]]. Given their coexistence with a diverse range of beneficial and pathogenic microorganisms that populate their gut [1,4], studies on shrimp microbiota management hold promise for enhancing shrimp survival and overall health [5,6]. Research about the intestinal microbiota of *P. vannamei* has been directed towards probiotic discovery and the understanding of pathogen-host-microbiome interactions [7]. For instance, prior studies have pinpointed Flavobacteriaceae and Rhodobacteraceae as potential probiotics due to their prevalence and abundance in healthy shrimp, whereas the family Rickettsiaceae and genus *Tenacibaculum* have been considered as “disease indicators' due to their abundance atrophying the hepatopancreas, causing a white opaque appearance of the muscle and reducing the body size of the shrimp [8].

*Vibrio* is a ubiquitous genus that tends to colonize the *P. vannamei* microbiome in freshwater, estuarine, and marine environments [9]. Some *Vibrio* strains can cause diseases by disrupting regular intestinal microbiota, leading to a microbial imbalance that facilitates disease progression and ultimately results in the death of the host [2]. One of the most serious diseases caused by *Vibrio* pathogenic species is the acute hepatopancreatic necrosis disease (AHPND) which carries a PVA plasmid with two homologous *PirAB* toxin genes [10]. AHPND-causing bacteria that proliferate in the gastrointestinal tract of shrimp alter microbial commensal functions, the immune system, and other important normal biological functions in the host [10,11].

The genera *Vibrio*, *Chlamydia*, *Rhodobacter*, *and Faecalibacterium*, among others, are significantly abundant in the entire gastrointestinal tract of juvenile *P. vannamei* shrimp affected by AHPND in an observational study [[11], [12], [13]]. This implies that the presence of a particular bacterial consortium in the gastrointestinal tract of shrimp may serve as an important indicator of disease. However, it is crucial to recognize that bacterial abundance and diversity within the entire gastrointestinal tract may vary across different organs [13,14]. This means that while *Vibrio* is a valuable signature, its abundance and distribution may not be consistent throughout the shrimp's gastrointestinal tract. Furthermore, there has been limited exploration of bacterial signatures within the stomachs and intestines when comparing healthy and AHPND-affected shrimp. This knowledge gap highlights the necessity for further research in this area to understand host dependence when AHPND occurs naturally. Similarly, the differentiation of niche-specific microbial signatures within various organs is needed for the correct use of disease bacterial biomarkers. Such insights can provide a more nuanced understanding of AHPND and potentially lead to more targeted interventions and management strategies.

In this study, we conducted a comprehensive microbiome analysis on juvenile *P. vannamei* shrimp from a commercial farm using high-throughput sequencing (HTS), with a focus on providing a clinically comparative framework of bacterial in the stomach and intestine of healthy and AHPND-affected shrimp. Our findings have revealed a profound disparity in both the bacterial diversity of the stomach and intestine microbiome between these two distinct health conditions. We have also identified bacterial signatures distinguishing healthy shrimp from those affected by AHPND. This newfound provides valuable insights into the intricate interplay of the shrimp microbiome under these conditions, enhancing our understanding of AHPND progression, and identification of probiotics tailored for the effective management of this disease.

## 2 Materials and methods

### 2.1 Sample collection and processing

**Fig. 1:**
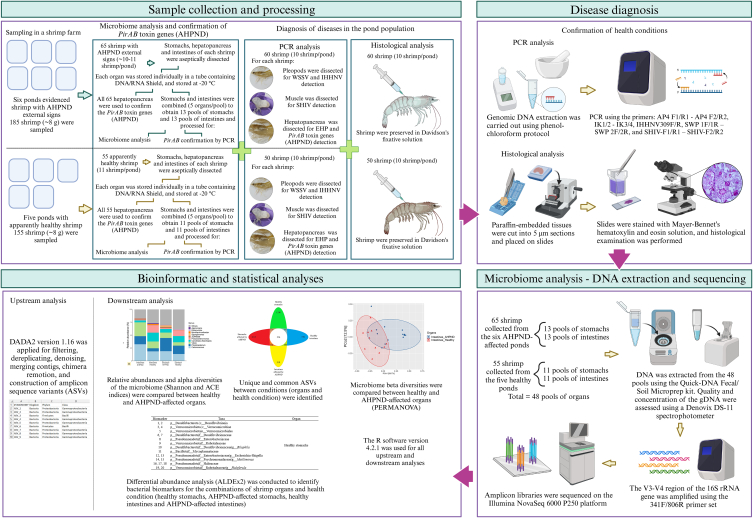
Experimental design for the analysis of bacterial communities in the stomach and intestine of *P. vannamei* juvenile shrimp affected by AHPND. Figure created with BioRender.com.

A total of 340 juvenile *P. vannamei* shrimp (∼8 g) were collected from 11 earthen ponds of a South American commercial farm (Fig. 1). Of these, six ponds evidenced shrimp with external signs of AHPND at the time of sampling. Subsequently, 185 shrimp with external signs of AHPND (empty intestine, pale hepatopancreas, and lethargy) were sampled from these six ponds. The remaining five ponds had shrimp that appeared healthy at the time of sampling. Subsequently, 155 apparently healthy shrimp (∼8 g) were sampled from these five ponds.

From the first group of 185 shrimp with external signs of AHPND, 65 shrimp (10–11 shrimp/pond) were used for the microbiome analysis and confirmation of *PirAB* toxin genes (AHPND) by PCR analysis (Fig. 1). The hepatopancreas, stomach, and intestine of each shrimp were aseptically dissected, and each organ was individually stored in a 1.5 mL microcentrifuge tube containing DNA/RNA Shield (Zymo Research, Irvine, CA, USA) at −20 °C. For confirmation of *PirAB* toxin genes by PCR analysis, each one of the 65 hepatopancreases was analyzed individually. Stomachs and intestines were combined to obtain pools of five shrimp organs, resulting in 13 pools of stomachs and 13 pools of intestines, and processed for confirmation of *PirAB* toxin genes by PCR analysis. In total, 26 pools were obtained in this form. This provided sufficient biomass for DNA extraction of each organ type.

At the same time, another 60 shrimp (10 shrimp/pond) from this first group of 185 shrimp were allocated for PCR analysis. Pleopods were dissected from each shrimp for detection of white spot syndrome virus (WSSV) and infectious hypodermal and hematopoietic necrosis virus (IHHNV). A segment of muscle was also dissected from each animal for shrimp hemocyte iridescent virus (SHIV) detection, and the hepatopancreas was dissected for detection of *Enterocytozoon hepatopenaei* (EHP) and *PirAB* toxin genes (AHPND). The remaining 60 shrimp (10 shrimp/pond) were preserved in Davidson's fixative solution to assess the health status by histological analysis.

From the second group of 155 apparently healthy shrimp, 55 shrimp (11 shrimp/pond) were used for the microbiome analysis and confirmation of *PirAB* toxin genes (AHPND) by PCR analysis (Fig. 1). The hepatopancreas, stomach, and intestine of each shrimp were also aseptically dissected, and each organ was individually stored in a 1.5 mL microcentrifuge tube containing DNA/RNA Shield (Zymo Research, Irvine, CA, USA) at −20 °C. For confirmation of *PirAB* toxin genes by PCR analysis, each one of the 55 hepatopancreases was analyzed individually. Stomachs and intestines were combined to obtain pools of five shrimp organs, resulting in 11 pools of stomachs and 11 pools of intestines, and processed for confirmation of *PirAB* toxin genes by PCR analysis. In total, 22 pools were obtained in this form. This provided sufficient biomass for DNA extraction of each organ type.

At the same time, another set of 50 shrimp (10 shrimp/pond) from the second group of 155 shrimp was allocated for PCR analysis. Organ processing for PCR analysis was the same as described for the first group of sampled shrimp. The remaining 50 shrimp (10 shrimp/pond) were preserved in Davidson's fixative solution to assess the health status by histological analysis.

### 2.2 Disease diagnosis

Genomic DNA (gDNA) extraction was carried out using the phenol-chloroform protocol (Fig. 1). The presence of *PirAB* toxin genes was assessed by nested PCR using the AP4 F1/R1 - AP4 F2/R2 primers [15]. WSSV, IHHNV, EHP, and SHIV infections were diagnosed using the IK1/2 - IK3/4 [4], IHHNV309 F/R [16], SWP 1 F/1 R–SWP 2 F/2 R [17], and SHIV–F1/R1 – SHIV–F2/R2 [18] primers, respectively. Positive and negative controls were used for each corresponding diagnosis. Histological analysis was carried out according to a published protocol [19]. Paraffin-embedded tissues were cut into 5-μm sections and placed on slides. The tissues were stained with Mayer-Bennet hematoxylin and eosin solutions for histological examination [19].

### 2.3 Microbiome analysis – DNA extraction and sequencing

The gDNA was extracted from the 48 pools using the Quick-DNA Fecal/Soil Microprep kit (Zymo Research, Irvine, CA, USA) following the manufacturer's instructions. The quality and concentration of the gDNA were assessed using a Denovix DS-11 spectrophotometer (Denovix Inc., USA). The V3–V4 region of the *16S rRNA* gene was amplified using the 341 F/806 R primer set [20]. Amplicon libraries were sequenced on the Illumina NovaSeq 6000 P250 platform by Novogene Incorporation (Sacramento, USA). The resulting sequences were deposited at the National Center of Biotechnology Information (NCBI) Sequence Read Archive (SRA), under BioProject accession number PRJNA947186.

### 2.4 Bioinformatic and statistical analyses

For the upstream analysis, DADA2 version 1.16 [21] was applied for filtering, dereplicating, denoising, merging contigs, chimera remotion, and construction of amplicon sequence variants (ASVs) (Fig. 1). The table of ASVs was taxonomically assigned using the SILVA 16 S rRNA database version 138.1. The resulting output files (taxonomy, ASVs, and metadata tables) were combined into a phyloseq object, and a microtable object was created using the microeco package version 0.13.0 [22]. Archaea, chloroplasts, mitochondria, and other sequences not related to bacteria were removed. The ASV table was then normalized using cumulative sum scaling (CSS) for the downstream analysis.

For the downstream analysis (Fig. 1), the microbiome alpha diversities [Shannon and abundance-based coverage estimator – (ACE) indices] were compared between healthy stomachs, AHPND-affected stomachs, healthy intestines, and AHPND-affected intestines using the Kruskal Wallis test. Unique (healthy stomachs, AHPND-affected stomachs, healthy intestines, and AHPND-affected intestines) and common (common to each shrimp organ without distinguishing the health condition, common to both shrimp organs for each health condition, and core common) ASVs were identified through a Venn diagram using the trans_venn function. The relative abundance at the phylum, order, family, and genus level was calculated for each case. For the microbiome beta diversity analysis, the Bray-Curtis index for the microbiome of healthy stomachs, AHPND-affected stomachs, healthy intestines, and AHPND-affected intestines was visualized with a principal coordinate analysis (PCoA). Differences in the microbiome between the stomachs of the two health conditions, as well as the intestines, and also between the stomachs and intestines in each health condition were conducted through a PERMANOVA analysis. For the differential abundance, the ANOVA-Like Differential Expression tool (ALDEx2) analysis for compositional data [[23], [24], [25], [26]] was used to find signatures between the microbiomes of healthy stomachs, AHPND-affected stomachs, healthy intestines, and AHPND-affected intestines. Concentric logarithmic ratio (clr) conversion to convert the abundance count data, the Kruskal Wallis test, and the interquartile logratio transformation (iqlr) were set as arguments into the function of ALDEx2.

## 3 Results

### 3.1 Disease diagnosis

The *PirAB* toxin genes were detected by PCR in each one of the 65 hepatopancreas of the sampled shrimp (six ponds) with external signs of AHPND (Supplementary Fig. 1) and in the corresponding 13 pools of intestines and 13 pools of stomachs conformed from these 65 shrimp, which were subsequently used for microbiome analysis (Supplementary Fig. 2). Meanwhile, the 55 hepatopancreas of the apparently healthy shrimp from the other five ponds (Supplementary Fig. 3) and the corresponding 11 pools of intestines and 11 pools of stomachs conformed from these 55 shrimp, which were also subsequently used for microbiome analysis (Supplementary Fig. 4), tested negative for the *PirAB* toxin genes by PCR.

The other 60 shrimp collected from the six ponds with external signs of AHPND tested positive for the *PirAB* toxin genes and were negative for the pathogenic genes involved in the most common shrimp diseases (WSSV, IHHNV, SHIV, and EHP). Meanwhile, the other 50 shrimp collected from the five ponds with apparently healthy shrimp tested negative for *PirAB* toxin genes and for the rest of the pathogenic genes of other shrimp diseases.

Additionally, signs of these diseases (WSSV, IHHNV, SHIV, and EHP) were not observed in any shrimp analyzed by histology.

**Fig. 2:**
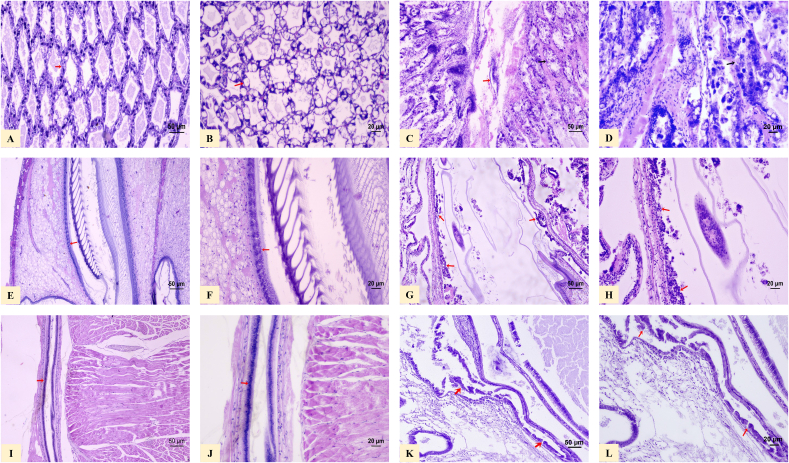
Histological sections of juvenile *P. vannamei* shrimp stained with hematoxylin and eosin solutions. The structure of the hepatopancreas tubules is observed under normal conditions (red arrow) at 4*x***(A)** and 10*x***(B)**. Severe necrosis of the hepatopancreas tubules (red arrow), characteristic of the terminal phase of acute hepatopancreatic necrosis disease (AHPND) at 4*x***(C)** and 10*x***(D)**. The structure of the stomach epithelium is observed under normal conditions (red arrow) at 4*x***(E)** and 10*x***(F)**. Detachment of the epithelial cells of the stomach, severe necrosis, and infiltration of hemocytes produced by AHPND (red arrow) at 4*x***(G)**, and 10*x***(H)**. The epithelium of the intestine is observed in normal conditions (red arrow) at 4*x***(I)** and 10*x***(J)**. Detachment of the epithelial cells of the intestine, severe necrosis, and infiltration of hemocytes produced by AHPND (red arrow) at 4*x***(K)** and 10*x***(L)**.

All 50 apparently healthy shrimp sampled for histological analysis exhibited normal cellular structures in the hepatopancreas, stomachs, and intestines (Fig. 2A, C, 2E). In contrast, all 60 shrimp with external signs of AHPND showed severe necrosis in the hepatopancreatic tubules, a typical lesion for the terminal phase of AHPND (Fig. 2B). Additionally, detachment of the epithelial cells, severe necrosis, and infiltration of hemocytes produced by AHPND were also observed in the stomachs and intestines of this group (Fig. 2D and F).

### 3.2 Bioinformatic and statistical analysis

After taxonomical assignment, a total of 4408 ASVs were identified at the lowest taxonomic level available in the 48 pools of organs. The Good's coverage showed an average of over 99 % for each sample.

#### 3.2.1 Microbiome alpha diversities

The microbiome alpha diversity indices were not significantly different when healthy stomachs, AHPND-affected stomachs, healthy intestines, and AHPND-affected intestines were compared (*P* = 0.184 and *P* = 0.156 for the Shannon and ACE indexes).

#### 3.2.2 Unique and common ASVs

**Fig. 3:**
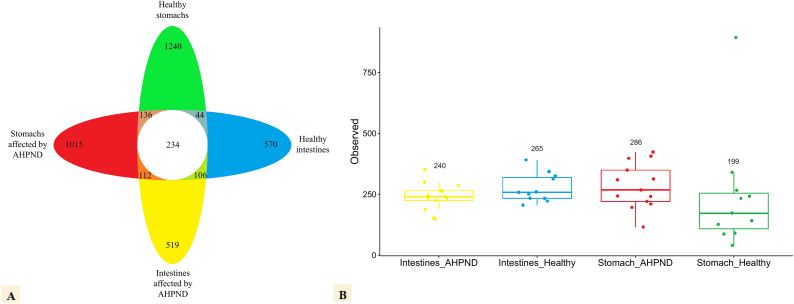
**(A).** Unique and common number of bacterial ASVs present in the stomachs and intestines of healthy and AHPND-affected *P. vannamei* shrimp. **(B)** Average of the ASVs observed in each of the samples.

The Venn diagram revealed unique and common ASVs in healthy and AHPND-affected organs (Fig. 3). Healthy stomachs showed the highest number of unique ASVs (1240, Fig. 3), among which only 2.0, 1.0, and 0.5 % belong to *Vibrio*, *Catenococcus,* and *Photobacterium*, respectively (Supplementary Table 1). Similarly, the second highest number of unique ASVs (1015) were found in stomachs affected by AHPND, among which only 2.0, 1.2, and 1.0 % belong to *Vibrio*, *Catenococcus,* and *Photobacterium*, respectively (Fig. 3).

Healthy and AHPND-affected intestines showed a similar amount of unique ASVs (570 and 519, respectively, Fig. 3). In healthy intestines, *Catenococcus* (2 %) and *Pseudomonas* (1.2 %) were the most abundant genera, while *Vibrio* (4 %), *Catenococcus* (4 %), and *Pseudomonas* (3 %) were abundant in AHPND-affected intestines (Supplementary Table 1).

On the other hand, after examination of the microbial composition of healthy and AHPND-affected stomachs, 136 common ASVs were found (Fig. 3). Within this common pool, *Pseudomonas* (8 %), *Acinetobacter* (7 %), and *Parabacteroides* (6 %) were the most abundant genera (Supplementary Table 1), while the genera *Vibrio*, *Catenococcus* and *Photobacterium* were each represented with an abundance of 0.7 % (Supplementary Table 1).

The microbiota of healthy and AHPND-affected intestines had 106 common ASVs (2.4 % = 106/4408, Fig. 3). Among them, the genera *Vibrio* (4 %), *Agarivorans* (4 %), *Catenococcus* (4 %), *Pseudomonas* (3 %), and *Photobacterium* (1.7 %) were the most abundant (Supplementary Table 1).

A total of 44 and 112 ASVs were common to organs of healthy and AHPND-affected shrimp, respectively (Fig. 3). In healthy organs, *Pseudoalteromonas* (11.3 %) and *Catenococcus* (2.3 %) were the most abundant genera, while the genera *Vibrio* (7 %) and *Catenococcus* (3 %) were abundant in organs affected by AHPND (Supplementary Table 1).

A total of 234 ASVs were common at both organs from both conditions and therefore were considered the core microbiome for the shrimp gastrointestinal tract (Fig. 3). *Vibrio* (14 %) was the most abundant genus among the common core, followed by *Catenococcus* (6 %), *Pseudoalteromonas* (4 %), *Photobacterium* (3 %), *Noviherbaspirillum* (2 %), *Pseudomonas* (2 %), and others ASVs represented 69 % (Supplementary Table 1).

#### 3.2.3 Relative abundance

**Fig. 4:**
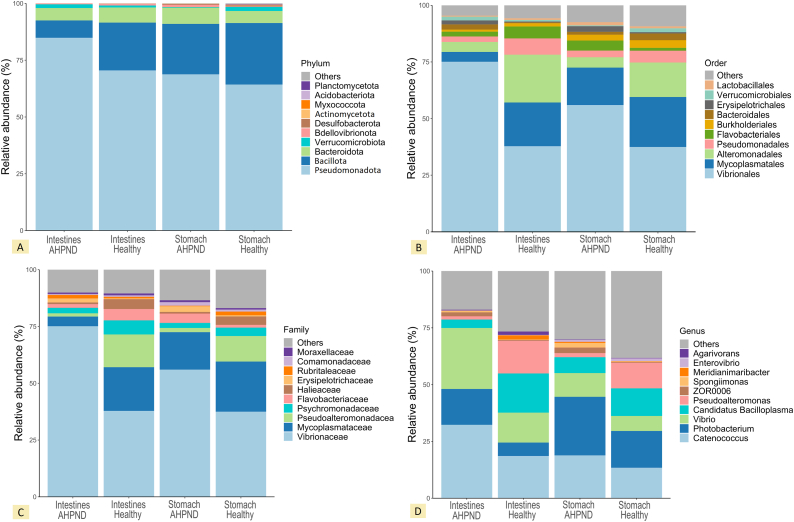
Relative abundance of bacterial communities in stomachs and intestines of healthy and AHPND-affected *P. vannamei* shrimp. Taxonomic diversity is described at the levels of: (**A**) Phylum; (**B**) Order; (**C**) Family; and (**D**) Genus.

The microbiome of healthy shrimp was dominated by three phyla: Pseudomonadota, Bacillota, and Bacteroidota, comprising 64.3 %, 27.1 %, and 5.3 % of the microbiota in the stomach, and 70.5 %, 21.1 %, and 6.7 % in the intestines, respectively (Fig. 4A and Supplementary Table 2). However, in shrimp affected by AHPND, the proportions shifted. In the stomachs of diseased shrimp, Pseudomonadota, Bacillota, and Bacteroidota accounted for 68.8 %, 22.2 %, and 7.1 %, respectively, while in the diseased intestines, they constituted 84.9 %, 7.6 %, and 5.4 %, respectively (Fig. 4A and Supplementary Table 2).

At the order level, Vibrionales predominated with 37.5 % in healthy stomachs, 37.8 % in healthy intestines, 56.0 % in AHPND-affected stomachs, and 75.1 % in AHPND-affected intestines. In both healthy and affected stomachs, Mycoplasmatales was the second most abundant order, representing 22.0 % and 16.5 %, respectively. However, its occurrence differed between healthy and affected intestines, constituting 19.2 % and 4.2 %, respectively. In contrast, Alteromonadales was the second most abundant family in both healthy and affected intestines, representing 21.1 % and 4.5 %, respectively (Fig. 4B and Supplementary Table 2).

The Vibrionaceae family exhibited the highest abundance across all organ samples under both health conditions (Fig. 4C and Supplementary Table 2). This family constituted 37.5 % of the ASVs found in healthy stomachs, 37.8 % in healthy intestines, 56.0 % in AHPND-infected stomachs, and a remarkable 75.1 % in AHPND-affected intestines (Fig. 4C and Supplementary Table 2). In all organ samples, Mycoplasmataceae predominated as the second most abundant family and was observed to increase in healthy organs. On the other hand, Pseudoalteromonadaceae was the third most abundant family with an abundance of 11.2 % and 14.4 % in healthy stomachs and intestines, respectively, while only 2.0 % and 1.4 % were present in stomachs and intestines affected by AHPND (Fig. 4C and Supplementary Table 2).

In healthy stomachs, the genera *Photobacterium*, *Catenococcus*, *Pseudoalteromonas*, *Vibrio*, and *Candidatus Bacilloplasma* accounted for abundances of 16.2 %, 13.4 %, 11.2 %, 6.6 %, and 12.1 %, respectively. In healthy intestines, these genera exhibited abundances of 5.9 %, 18.6 %, 14.4 %, 13.2 %, and 17.2 % (Fig. 4D and Supplementary Table 2). In stomachs affected by AHPND, the abundances were 25.8 %, 18.8 %, 1.8 %, 10.5 %, and 6.9 % for *Photobacterium*, *Catenococcus*, *Pseudoalteromonas*, *Vibrio*, and *Candidatus Bacilloplasma*, respectively (Fig. 4D and Supplementary Table 2). Conversely, in intestines affected by AHPND, the abundances were 15.8 %, 32.3 %, 1.4 %, 26.8 %, and 3.7 %, respectively (Fig. 4D and Supplementary Table 2).

#### 3.2.4 Microbiome beta diversities

**Fig. 5:**
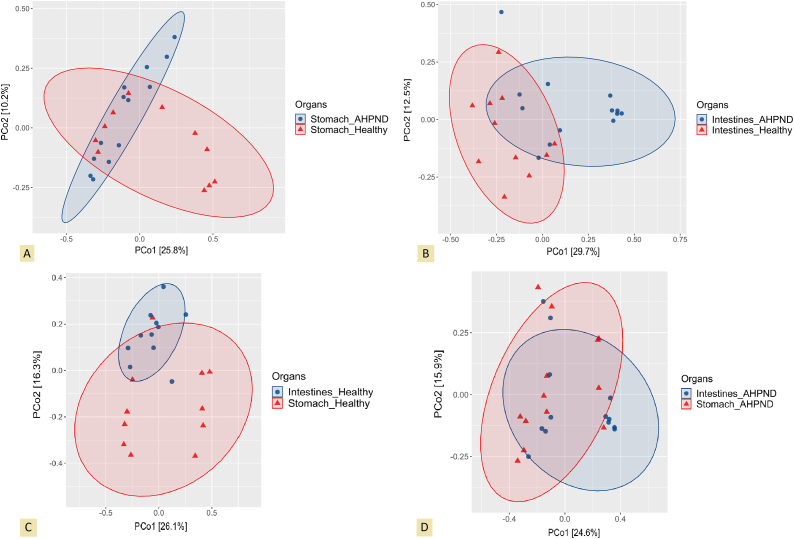
Principal coordinate analysis (PCoA) based on the Bray-Curtis index shows the differences of bacterial communities between healthy and AHPND-affected stomachs (**A**) and intestines (**B**) of *P. vannamei*. Furthermore, the comparison is drawn in (**C**) between healthy intestines and healthy stomachs, and in (**D**) between intestines and stomachs affected by AHPND.

PERMANOVA analysis revealed remarkable dissimilarities in the beta diversity of bacterial communities of stomachs and intestines (*P* = 0.003), irrespective of health condition. The Bray-Curtis index of bacterial communities showed significant disparities in the microbial beta diversity of healthy and AHPND-affected stomachs (*P* = 0.017, Fig. 5A), and of healthy and AHPND-affected intestines (*P* = 0.001, Fig. 5B). Furthermore, a significant divergence emerged in the comparison between healthy intestines and healthy stomachs (*P* = 0.002, Fig. 5C), whereas no significant differences were observed between diseased intestines and diseased stomachs (*P* = 0.080, Fig. 5D).

#### 3.2.5 Differential abundance analysis

A total of 53 ASV signatures were identified when healthy stomachs, AHPND-affected stomachs, healthy intestines, and AHPND-affected intestines were compared (ALDEx2 iqlr, *P* < 0.05, Table 1). Of these, 23 ASVs were found in healthy stomachs, including nine ASVs in the Pseudomonadota phylum, six ASVs each in the Verrucomicrobiota and Desulfobacterota phyla, and two in the Bacillota phylum. Some genera such as *Bilophila*, *Escherichia-Shigella*, *Pseudoalteromonas*, *Motilimonas*, and *Haloferula* were significantly more abundant in healthy shrimp stomachs (Table 1). In contrast, only two ASVs belonging to the Gammaproteobacteria class were significantly abundant in AHPND-affected stomachs (Table 1).

**Table 1:** Results of the differential abundance analysis using ALDEx2 iqrl for the comparison between healthy stomachs, AHPND-affected stomachs, healthy intestines, and AHPND-affected intestines. ASVs with *P* < 0.05 were considered bacterial signatures.

No. Of signature	Taxa	Organ
1, 2	p__Desulfobacterota |c__Desulfovibrionia	Healthy stomachs
3, 4	p__Verrucomicrobiota |c__Verrucomicrobiae	
5	p__Verrucomicrobiota|o__Verrucomicrobiales	
6, 7	p__Desulfobacterota|f__Desulfovibrionaceae	
8	p__Pseudomonadota|f__Enterobacteriaceae	
9	p__Verrucomicrobiota|f__Rubritaleaceae	
10	p__Desulfobacterota|f__Desulfovibrionaceae|g__*Bilophila*	
11	p__Bacillota|f__Mycoplasmataceae	
12, 13	p__Pseudomonadota|f__Enterobacteriaceae|g__Escherichia-Shigella	
14, 15	p__Pseudomonadota|f__Psychromonadaceae|g__*Motilimonas*	
16, 17, 18	p__Pseudomonadota|f__Halieaceae	
19, 20	p__Verrucomicrobiota|f__Rubritaleaceae|g__*Haloferula*	
21	p__Desulfobacterota|f__Desulfovibrionaceae|g__*Bilophila|s__wadsworthia*	
22	p__Bacillota|f__Mycoplasmataceae	
23	p__Pseudomonadota|g__*Pseudoalteromonas|s__spongiae*	
24, 25	p__Pseudomonadota|c__Gammaproteobacteria	AHPND-affected stomachs
26	p__Pseudomonadota|f__Pseudoalteromonadaceae	Healthy intestines
27	p__Pseudomonadota|f__Cellvibrionaceae	
28, 29	p__Pseudomonadota|f__Pseudoalteromonadaceae|g__*Pseudoalteromonas*	
30	p__Pseudomonadota|f__Cellvibrionaceae|g__*Gilvimarinus*	
31, 32	p__Pseudomonadota|f__Halieaceae|g__*Parahaliea*	
33	p__Pseudomonadota|f__Psychromonadaceae|g__*Agarivorans*	
34	p__Pseudomonadota|f__Cellvibrionaceae|g__*Gilvimarinus*	
35	p__Bacteroidota|o__Bacteroidales	AHPND-affected intestines
36, 37	p__Pseudomonadota|c__Gammaproteobacteria	
38, 39, 40	p__Bacteroidota|f__Marinifilaceae	
41, 42	p__Bdellovibrionota|f__Bacteriovoracaceae|g__*Peredibacter*	
43, 44	p__Pseudomonadota|g__*Candidatus Berkiella*	
45, 46	p__Pseudomonadota|f__Alteromonadaceae|g__*Aestuariibacter*	
47, 48	p__Pseudomonadota|f__Moraxellaceae|g__*Acinetobacter*	
49	p__Verrucomicrobiota|f__Rubritaleaceae|g__*Roseibacillus*	
50	p__Bacteroidota|f__Marinilabiliaceae|g__*Labilibacter*	
51	p__Pseudomonadota|f__Vibrionaceae|g__*Vibrio|s__brasiliensis*	
52	p__Pseudomonadota|f__Vibrionaceae|g__*Vibrio|s__sinaloensis*	
53	p_Verrucomicrobiota|f_Rubritaleaceae|g_*Roseibacillus*	

Nine ASVs from four families, identified as Pseudoalteromonadaceae, Psychromonadaceae, Halieaceae, and Cellvibrionaceae were significantly abundant in healthy shrimp intestines (Table 1). Of these ASVs, five were identified to the genus level, including *Pseudoalteromonas*, *Agarivorans*, *Gilvimarinus*, and *Parahaliea*. ASVs belonging to Pseudoalteromonadaceae and Halieaceae were the most abundant among all bacterial signatures in healthy shrimp (Supplementary Fig. 5).

A total of 19 ASVs were significantly abundant in intestines affected by AHPND. Although the Vibrionaceae was the most relatively abundant family in all organs, irrespective of health condition, two ASVs belonging to Vibrionaceae (particularly the genera *Vibrio brasiliensis* and *Vibrio sinaloensis*) were significantly abundant in intestines affected by AHPND, followed by Bacteriovoracaceae, Alteromonadaceae, Marinifilaceae, and Rubritaleaceae (Table 1).

## 4 Discussion

The shrimp intestine is a metabolically active organ where several functional processes between the host and the microbiome take place [2]. The first barrier in the intestinal mucosa of shrimp is the epithelial cells, and its integrity is linked to the microbial community, which in turn is closely related to the health status [27]. When pathogenic vibrios invade the shrimp, there is a decline in the epithelial activity of the intestinal cells [27,28] and the bacterial networks are altered [11], leading to a reduction of intestinal immunity [28]. Therefore, the stability of the gut microbiome plays a key role in how a disease progresses in shrimp [27,29]. A specific example occurs when *P. vannamei* shrimp are exposed to colonization by *Vibrio alginolyticus*, as within 48 h of infection, changes in the gut microbiome are observed, especially a decrease in the presence of *Candidatus-Bacilloplasma*, marking the onset of disease [27].

In our study, the impact of AHPND was reflected in changes in the relative abundance of the microbiome. Thus, a clear significant difference in the microbial beta diversity, mostly marked in the intestinal microbiome, and bacterial signatures were observed when comparing the intestines and stomachs affected by AHPND.

The results of the beta diversity analysis in healthy shrimp suggest the existence of specific microbial niches in the stomach and intestine, a fact that is consistent with other studies [13,14]. However, interestingly, this differentiation disappeared in diseased shrimp, which exhibited no significant differences in microbiomes between these two organs. This observation could suggest a longitudinal homogenization of the gastrointestinal microbiome in response to disease [30]. Another possible interpretation is that when shrimp are diseased, their immune system or conditions in the gastrointestinal tract may change in a way that promotes increased microbial homogeneity rather than maintaining specialized niches in the stomachs and intestines. This uniformity could be a protective response, a way in which the organism attempts to stabilize the microbial ecosystem during the infection.

Several biomarkers associated with healthy conditions and AHPND disease have been proposed [6,12,13]. For example, *P. vannamei* shrimp experimentally challenged with AHPND-causing pathogenic *Vibrio* exhibit members of the order Vibrionales as biomarkers in the stomach microbiome [6], while the genera *Aeromonas*, *Simiduia,* and *Photobacterium* are biomarkers in the intestinal microbiome of shrimp naturally affected by AHPND [13]. However, these studies have been conducted under controlled conditions using a particular bacterial strain [6], have had a limited number of samples from naturally diseased shrimp [13], or analyzed the entire gastrointestinal tract, without distinguishing between organs [12]. Studies of organ-specific niches may also provide a more nuanced understanding of the shrimp microbiome response to colonization by AHPND-causing bacteria, and potentially lead to more successful interventions and management strategies.

In the present study, the Vibrionaceae family (Pseudomonadota member), which includes the three main genera *Catenococcus*, *Photobacterium,* and *Vibrio*, dominated the gastrointestinal of *P. vannamei* shrimp compared to all other bacterial genera in all shrimp. Vibrionaceae play primary roles in *P. vannamei* shrimp, specifically with the *Vibrio* genus, which interacts with the shrimp's immune system and adaptation to the environment [[31], [32], [33]]. This observation aligns with our study's findings, that identified *Vibrio* as the central component within the shrimp's gastrointestinal tract, which includes both the stomach and intestine.

*Catenococcus* is associated with the oxidation of sulfur compounds as a self-beneficial process [34,35]. Additionally, *Catenococcus*, *Photobacterium,* and *Vibrio* have been linked to AHPND pathogenicity. In a previous study, we found that *Catenococcus* is an AHPND biomarker in *P. vannamei* larvae [5], while *Photobacterium* is a biomarker associated with juvenile shrimp affected by vibriosis [13,36]. In this study, these genera were relatively abundant in organs affected by AHPND, but only *Vibrio brasiliensis* and *V. sinaloensis* were considered signatures associated with AHPND-affected intestines. These vibrios have been linked to high mortalities in juvenile *P. vannamei* [37], but no previous studies have shown a direct relationship between *V. brasiliensis* and *V. sinaloensis* with AHPND.

We also observed the presence of other members of Pseudomonadota, such as *Gilvimarinus*, in healthy intestines. *Gilvimarinus* has been found to exhibit positive interactions within bacterial networks of surviving *P. vannamei* after infection with *V. parahaemolyticus* causing AHPND [9]. Furthermore, the abundance of *Gilvimarinus* plays a key role in the immune response of *P*. *vannamei* shrimp infected with *Vibrio parahaemolyticus* [38]. Overall, these findings underscore the importance of understanding how *Gilvimarinus* may play a role in immune response and resistance to adverse health conditions.

Our study found a considerable increase in the family Pseudoalteromonadaceae, particularly its main genus *Pseudoalteromonas,* in healthy organs, including some ASV signatures that were more abundant than others, especially in healthy intestines of *P. vannamei* shrimp. Pseudoalteromonadaceae is well-known to play a beneficial role in regulating the immune system and producing chemical substances like proteases and antimicrobial compounds in *P. vannamei* shrimp [39,40]. *Pseudoalteromonas* has already been experimentally tested with juvenile *P. vannamei* shrimp infected by AHPND bacteria [41] and other pathogenic vibrios [42] and it increased shrimp survival after a few days of supplementation [41,42]. Our findings suggest that *Pseudoalteromonas* could be a specific probiotic for the control of AHPND. Moreover, our results indicate that multiple ASVs or taxa within the *Pseudoalteromonadaceae* family may contribute to the antagonism against AHPND.

Verrucomicrobiota and Desulfobacterota were the second phyla with more signatures in healthy *P. vannamei* stomachs in our study. Members of the Verrucomicrobiota have been reported as beneficial in assisting digestive processes, such as polysaccharide degradation in aquatic species like sea cucumbers [43]. *Haloferula*, a member of Verrucomicrobiota, is a probiotic that reduces the abundance of pathogenic vibrios in juvenile sea cucumbers [43]. Desulfobacterota are bacteria that inhabit anaerobic conditions and participate in the metabolic processes of marine species [44]. The presence of the genus *Bilophila* (Desulfobacterota) has only been observed in the intestine of *P. vannamei* treated with a probiotic strain of *Bacillus subtilis* [45]*,* and its role in the shrimp microbiome is unclear. To the best of our knowledge, no reports have described the antagonism of *Haloferula* and *Bilophila* genera towards AHPND. Further isolation and study of these bacteria are recommended for practical purposes. *Roseibacillus* (another member of Verrucomicrobiota) was observed in the microbiome of *P. monodon* affected by *Vibrio harveyi* [46], whereas, in our study, *Roseibacillus* was a signature of AHPND-affected intestines. This finding suggests that, like *Pseudomonadota*, some members of Verrucomicrobiota can be both beneficial and pathogenic to farmed shrimp.

Although the individuals used to confirm infection with AHPND-causing bacteria were the same individuals used for the microbiome analysis, a limitation of this study is that different shrimp were used for the histological analysis. The latter analysis requires all intact organs of the gastrointestinal tract (hepatopancreas, stomach, and intestine), as the whole animal is preserved and processed for lesion observation. However, given that in the group of 185 shrimp with external signs of AHPND, all 60 shrimp processed for histology were positive for the disease and showed lesions typical of the terminal phase of AHPND (severe necrosis in the hepatopancreatic tubules and epithelial cell sloughing with necrosis and hemocyte infiltration in the stomach and intestines), the disease prevalence in this sample was 100 % (60 shrimp positive for AHPND/60 shrimp processed for histology). This means that the probable prevalence of AHPND disease in all 185 shrimp sampled was 100 %, with little or no error. The same logic applies to the second group of 155 apparently healthy shrimp, as all 60 histologically processed shrimp in this group were negative for the disease.

## 5 Conclusions

In this study, we conducted a comprehensive microbiome analysis on juvenile *P. vannamei* shrimp from a commercial farm using high-throughput sequencing (HTS), with a focus on providing a clinically comparative framework of bacterial in the stomach and intestine of healthy and AHPND-affected shrimp. Our investigation revealed a profound disparity in both the diversity of the stomach and intestine microbiome between these two distinct health conditions. Notably, we identified a total of 53 signatures that exhibited significant differences between healthy and AHPND-affected shrimp, including the discovery of bacterial taxa associated with both health states. These novel findings explain the intricate interplay within the microbiome of healthy and AHPND-affected shrimp, providing valuable insights into the dynamics and progression of AHPND. Furthermore, our findings hold promise for advancing the development of probiotic interventions aimed at mitigating AHPND. Although we are far from developing shrimp probiotics to prevent or mitigate the effects of AHPND with precision approaches using HTS technology (*16S rRNA*), the identification of different bacterial taxa in the healthy stomachs and intestines compared to AHPND-affected stomachs and intestines can be refined with more sophisticated data analysis (shotgun metagenome) and culturomics techniques to attempt to isolate potential probiotic strains in dependent culture samples, guided by the information of specific bacterial signatures identified in the microbiome analysis. The same approach can be used to deepen the bacterial signatures that appear to co-occur with AHPND-causing bacteria. In the future, more data and laboratory analysis will be needed to find practical solutions for shrimp disease management.

## Funding

This research was founded by the Secretaría de Educación Superior, Ciencia, Tecnología e Innovación (SENESCYT) of Ecuador in the framework of the Inédita project N° PIC-21-INE-ESPOL-004, “Biotecnología azul para el fortalecimiento de la industria acuícola ecuatoriana controlando Vibrios patógenos”.

## Ethics statement

No ethical approval was required for this study. However, in order to ensure ethical treatment, a procedure involving inmersion in 60 % alcohol was used to euthanize the animals.

## Data availability statement

The resulting sequences were deposited at the National Center of Biotechnology Information (NCBI) Sequence Read Archive (SRA), under BioProject accession number PRJNA947186.

## CRediT authorship contribution statement

**Guillermo Reyes:** Writing – review & editing, Writing – original draft, Visualization, Validation, Software, Methodology, Investigation, Formal analysis, Data curation, Conceptualization. **Betsy Andrade:** Writing – review & editing, Visualization, Methodology, Investigation, Formal analysis. **Irma Betancourt:** Writing – review & editing, Methodology, Investigation, Formal analysis. **Fanny Panchana:** Writing – review & editing, Visualization, Methodology, Formal analysis. **Cristhian Preciado:** Writing – review & editing, Methodology, Formal analysis. **Bonny Bayot:** Writing – review & editing, Validation, Supervision, Resources, Project administration, Investigation, Funding acquisition, Data curation, Conceptualization.

## Declaration of competing interest

The authors declare that they have no known competing financial interests or personal relationships that could have appeared to influence the work reported in this paper.

## References

[1] Zheng Y., Yu M., Liu J., Qiao Y., Wang L., Li Z., Zhang X., Yu M. (2017). Bacterial community associated with healthy and diseased Pacific white shrimp (Litopenaeus vannamei) larvae and rearing water across different growth stages. Front. Microbiol..

[2] Dai W., Sheng Z., Chen J., Xiong J. (2020). Shrimp disease progression increases the gut bacterial network complexity and abundances of keystone taxa. Aquaculture.

[3] Zhang D., Wang X., Xiong J., Zhu J., Wang Y., Zhao Q., Chen H., Guo A., Wu J., Dai H. (2014). Bacterioplankton assemblages as biological indicators of shrimp health status. Ecol. Indicat..

[4] Hossain Md S., Otta S.K., Chakraborty A., Sanath Kumar H., Karunasagar I., Karunasagar I. (2004). Detection of WSSV in cultured shrimps, captured brooders, shrimp postlarvae and water samples in Bangladesh by PCR using different primers. Aquaculture.

[5] Reyes G., Betancourt I., Andrade B., Panchana F., Román R., Sorroza L., Trujillo L., Bayot B. (2022). Microbiome of Penaeus vannamei larvae and potential biomarkers associated with high and low survival in shrimp hatchery tanks affected by acute hepatopancreatic necrosis disease. Front. Microbiol..

[6] Restrepo L., Domínguez-Borbor C., Bajaña L., Betancourt I., Rodríguez J., Bayot B., Reyes A. (2021). Microbial community characterization of shrimp survivors to AHPND challenge test treated with an effective shrimp probiotic (Vibrio diabolicus). Microbiome.

[7] Li E., Xu C., Wang X., Wang S., Zhao Q., Zhang M., Qin J., Chen L. (2018). Gut microbiota and its modulation for healthy farming of pacific white shrimp Litopenaeus vannamei. Reviews in Fisheries Science & Aquaculture.

[8] Zhou L., Chen C., Xie J., Xu C., Zhao Q., Qin J., Chen L., Li E. (2019). Intestinal bacterial signatures of the ‘cotton shrimp-like’ disease explain the change of growth performance and immune responses in Pacific white shrimp (Litopenaeus vannamei). Fish Shellfish Immunol..

[9] Alvarez-Ruiz S., Luna-González A., Escamilla-Montes R., Fierro-Coronado A., Diarte-Plata G., García-Gutiérrez C., Peraza-Gómez V. (2022). Gut bacterial profile associated with healthy and diseased (AHPND) shrimp Penaeus vannamei. Lat Am J Aquat Res.

[10] Han J., Tang K., Tran L., Lightner D. (2015). Photorhabdus insect-related (Pir) toxin-like genes in a plasmid of Vibrio parahaemolyticus, the causative agent of acute hepatopancreatic necrosis disease (AHPND) of shrimp. Dis. Aquat. Org..

[11] Chen W.Y., Ng T.H., Wu J.H., Chen J.W., Wang H.C. (2017). Microbiome dynamics in a shrimp grow-out pond with possible outbreak of acute hepatopancreatic necrosis disease. Sci. Rep..

[12] Dong P., Guo H., Wang Y., Wang R., Chen H., Zhao Y., Wang K., Zhang D. (2021). Gastrointestinal microbiota imbalance is triggered by the enrichment of Vibrio in subadult Litopenaeus vannamei with acute hepatopancreatic necrosis disease. Aquaculture.

[13] Cornejo-Granados F., Lopez-Zavala A., Gallardo-Becerra L., Mendoza-Vargas A., Sánchez F., Vichido R., Sotelo-Mundo R., Ochoa-Leyva A. (2017). Microbiome of Pacific Whiteleg shrimp reveals differential bacterial community composition between Wild, Aquacultured and AHPND/EMS outbreak conditions. Sci. Rep..

[14] Garibay-Valdez E., Cicala F., Martinez-Porchas M., Gómez-Reyes R., Vargas-Albores F., Gollas-Galván T., Calderón K. (2021). Longitudinal variations in the gastrointestinal microbiome of the white shrimp, Litopenaeus vannamei. PeerJ.

[15] Dangtip S., Sirikharin R., Sanguanrut P., Thitamadee S., Sritunyalucksana K., Taengchaiyaphum S., Mavichak R., Proespraiwong P., Flegel T. (2015). AP4 method for two-tube nested PCR detection of AHPND isolates of Vibrio parahaemolyticus. Aquac Rep.

[16] Tang K., Navarro S., Lightner D. (2007). PCR assay for discriminating between infectious hypodermal and hematopoietic necrosis virus (IHHNV) and virus-related sequences in the genome of Penaeus monodon. Dis. Aquat. Org..

[17] Jaroenlak P., Sanguanrut P., Williams B., Stentiford G., Flegel T., Sritunyalucksana K., Itsathitphaisarn O. (2016). A nested PCR assay to avoid false positive detection of the microsporidian Enterocytozoon hepatopenaei (EHP) in environmental samples in shrimp farms. PLoS One.

[18] Qiu L., Chen M., Wan X., Li C., Zhang Q., Wang R., Cheng D., Dong X., Yang B., Wang X., Xiang J., Huang J. (2017). Characterization of a new member of Iridoviridae, Shrimp hemocyte iridescent virus (SHIV), found in white leg shrimp (Litopenaeus vannamei). Sci. Rep..

[19] T. Bell, D. V. Lightner, “A handbook of normal penaeid shrimp histology.” Accessed: September. 20, 2021. [Online]. Available: https://agris.fao.org/agris-search/search.do?recordID=XF2016018755.

[20] Takahashi S., Tomita J., Nishioka K., Hisada T., Nishijima M. (2014). Development of a prokaryotic universal primer for simultaneous analysis of bacteria and archaea using next-generation sequencing. PLoS One.

[21] Callahan B.J., McMurdie P.J., Rosen M.J., Han A.W., Johnson A.J.A., Holmes S.P. (2016). DADA2: high-resolution sample inference from Illumina amplicon data. Nat. Methods.

[22] Liu C., Cui Y., Li X., Yao M. (2021). Microeco : an R package for data mining in microbial community ecology. FEMS Microbiol. Ecol..

[23] Fernandes A.D., Reid J.N., Macklaim J.M., McMurrough T.A., Edgell D.R., Gloor G.B. (2014). Unifying the analysis of high-throughput sequencing datasets: characterizing RNA-seq, 16S rRNA gene sequencing and selective growth experiments by compositional data analysis. Microbiome.

[24] Cao Y., Dong Q., Wang D., Zhang P., Liu Y., Niu C. (2022). microbiomeMarker: an R/Bioconductor package for microbiome marker identification and visualization. Bioinformatics.

[25] Gloor G., Reid G. (2016). Compositional analysis: a valid approach to analyze microbiome high-throughput sequencing data. Can. J. Microbiol..

[26] Gloor G., Pistner N., Silverman J. (2023). Normalizations are not what you think; Explicit Scale Simulation in ALDEx2. bioRxiv.

[27] Liao G., Wu Q., Mo B., Zhou J., Li J., Zou J., Fan L. (2022). Intestinal morphology and microflora to Vibrio alginolyticus in pacific white shrimp (Litopenaeus vannamei). Fish Shellfish Immunol..

[28] Liang F., Li C., Hou T., Wen C., Kong S., Ma D., Sun C., Li S. (2020). Effects of chitosan–gentamicin conjugate supplement on non-specific immunity, aquaculture water, intestinal histology and microbiota of pacific white shrimp (Litopenaeus vannamei). Mar. Drugs.

[29] Kumar V., De Bels L., Couck L., Baruah K., Bossier P., Van Den Broeck W. (2019). PirABVP toxin binds to epithelial cells of the digestive tract and produce pathognomonic AHPND lesions in germ-free brine shrimp. Toxins.

[30] Belkaid Y., Hand T.W. (2014). Role of the microbiota in immunity and inflammation. Cell.

[31] Dong P., Guo H., Wang Y., Wang R., Chen H., Zhao Y., Wang K., Zhang D. (2021). Gastrointestinal microbiota imbalance is triggered by the enrichment of Vibrio in subadult Litopenaeus vannamei with acute hepatopancreatic necrosis disease. Aquaculture.

[32] Zhang X., Lin H., Wang X., Austin B. (2018). Significance of Vibrio species in the marine organic carbon cycle—a review. Sci. China Earth Sci..

[33] Wang H., Huang J., Wang P., Li T. (2020). Insights into the microbiota of larval and postlarval Pacific white shrimp (Penaeus vannamei) along early developmental stages: a case in pond level.

[34] Sorokin D. Yu, Robertson L.A., Kuenen J.G. (1996). Sulfur cycling in Catenococcus thiocyclus. FEMS Microbiol. Ecol..

[35] Patil P.K., Vinay T.N., Aravind R., Avunje S., Vijayan K.K. (2021). Effect of Bacillus spp. on the composition of gut microbiota in early life stages of Indian white shrimp, Penaeus indicus. J. Appl. Aquacult..

[36] Enciso-Ibarra J., González-Castillo A., Soto-Rodriguez S.A., Enciso-Ibarra K., Bolán-Mejia C., Gomez-Gil B. (2020). Photobacterium lucens sp. nov., isolated from a cultured shrimp Penaeus vannamei. Curr. Microbiol..

[37] Li G., Xie G., Wang H., Wan X., Li X., Shi C., Wang Z., Gong M., Li T., Wang P., Zhang Q., Huang J. (2021). Characterization of a novel shrimp pathogen, Vibrio brasiliensis , isolated from Pacific white shrimp, Penaeus vannamei. J. Fish. Dis..

[38] Lv X., Li S., Yu Y., Zhang X., Li F. (2020). Characterization of a gill-abundant crustin with microbiota modulating function in Litopenaeus vannamei. Fish Shellfish Immunol..

[39] Holmström C. (2002). Antifouling activities expressed by marine surface associated Pseudoalteromonas species. FEMS Microbiol. Ecol..

[40] Ray G.W., Liang D., Yang Q., Tan B., Dong X., Chi S., Liu H., Zhang S., Rimei L. (2020). Effects of replacing fishmeal with dietary soybean protein concentrate (SPC) on growth, serum biochemical indices, and antioxidative functions for juvenile shrimp Litopenaeus vannamei. Aquaculture.

[41] Wang H., Wang C., Tang Y., Sun B., Huang J., Song X. (2018). Pseudoalteromonas probiotics as potential biocontrol agents improve the survival of Penaeus vannamei challenged with acute hepatopancreatic necrosis disease (AHPND)-causing Vibrio parahaemolyticus. Aquaculture.

[42] Louis S., Wabete N., Ansquer D., Mailliez J., Pallud M., Zhang C., Lindivat M., Boulo V., Pham D. (2018). Survival improvement conferred by the Pseudoalteromonas sp. NC201 probiotic in Litopenaeus stylirostris exposed to Vibrio nigripulchritudo infection and salinity stress. Aquaculture.

[43] Li Y., Zhao Y., Liu X., Yuan L., Liu X., Wang L., Sun H. (2022). Effects of endogenous potential probiotic Lactobacillus rhamnosus M2-4 on intestinal microflora and metabonomics in juvenile sea cucumber Apostichopus japonicus. Aquaculture.

[44] Murphy C.L., Biggerstaff J., Eichhorn A., Ewing E., Shahan R., Soriano D., Stewart S., VanMol K., Walker R., Walters P., Elshahed M.S., Youssef N.H. (2021). Genomic characterization of three novel Desulfobacterota classes expand the metabolic and phylogenetic diversity of the phylum. Environ. Microbiol..

[45] Cheng A., Yeh S., Hu S., Lin H., Liu C. (2020). Intestinal microbiota of white shrimp, Litopenaeus vannamei, fed diets containing Bacillus subtilis E20‐fermented soybean meal (FSBM) or an antimicrobial peptide derived from B. subtilis E20‐FSBM. Aquacult. Res..

[46] Angthong P., Uengwetwanit T., Uawisetwathana U., Koehorst J.J., Arayamethakorn S., Schaap P.J., Martins Dos Santos V., Phromson M., Karoonuthaisiri N., Chaiyapechara S., Rungrassamee W. (2023). Investigating host-gut microbial relationship in Penaeus monodon upon exposure to Vibrio harveyi. Aquaculture.

