# Supplementary material for: Bacterial communities and signatures in the stomach and intestine of juvenile Penaeus (litopenaeus) vannamei shrimp affected by acute hepatopancreatic necrosis disease
Source: Heliyon. 2024 Jun 15;10(12):e33034. doi: 10.1016/j.heliyon.2024.e33034 (PMC11239581; doi:10.1016/j.heliyon.2024.e33034)
Supplement: Multimedia component 1 [file mmc1.pdf]

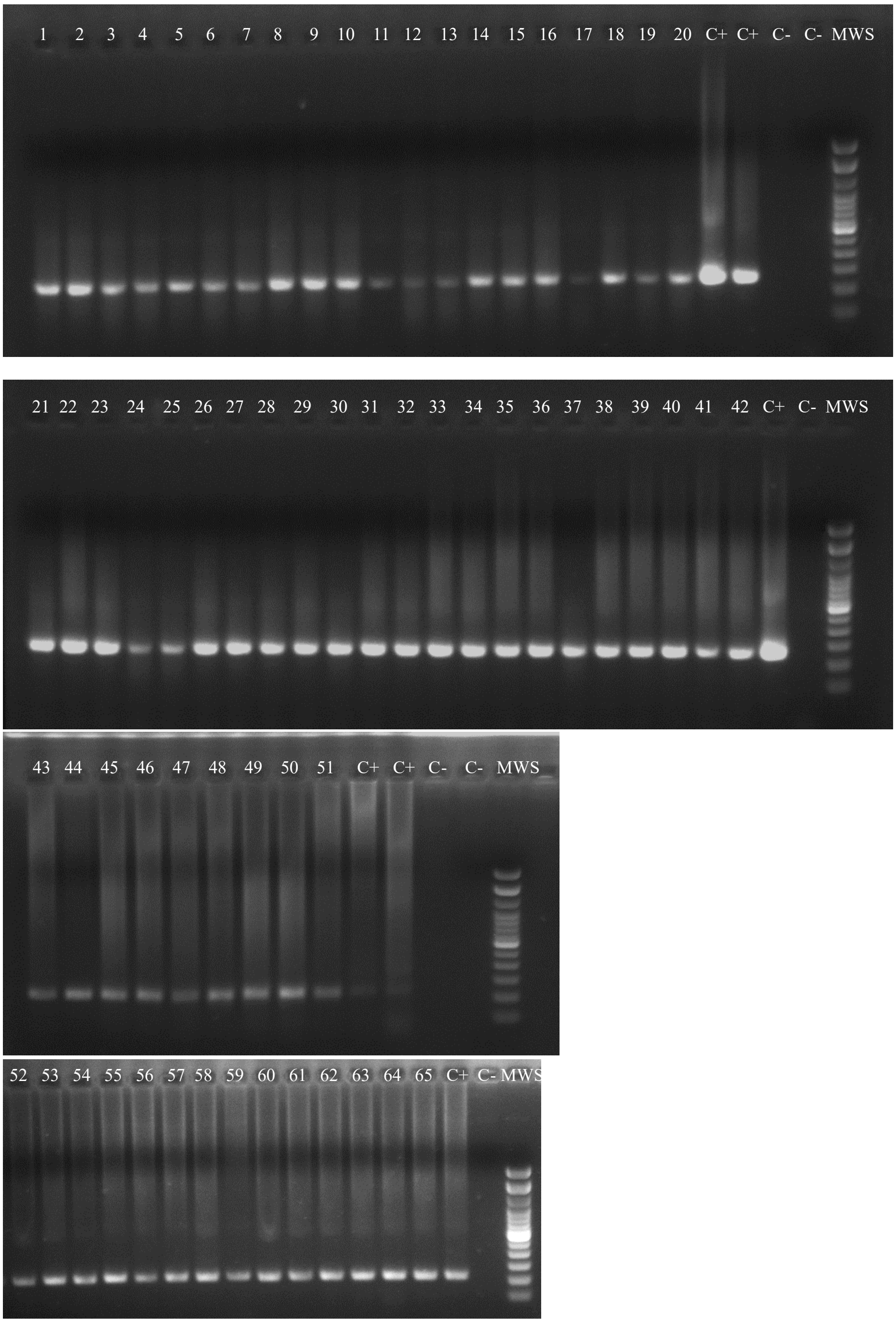

Supplementary Figure 1. Agarose gel of PCR products amplified using the specific primers for the detection of *PirAB* toxin genes (AHPND). PCR confirmation was conducted for hepatopancreas of 65 shrimp with external signs of AHPND, used for microbiome analysis. Positive controls (C+) and negative controls (C-) were processed. MWS = Molecular Weight Standard (Gene Ruler 100 bp DNA Ladder). For the positive controls, an amplified product of 230 bp is observed.

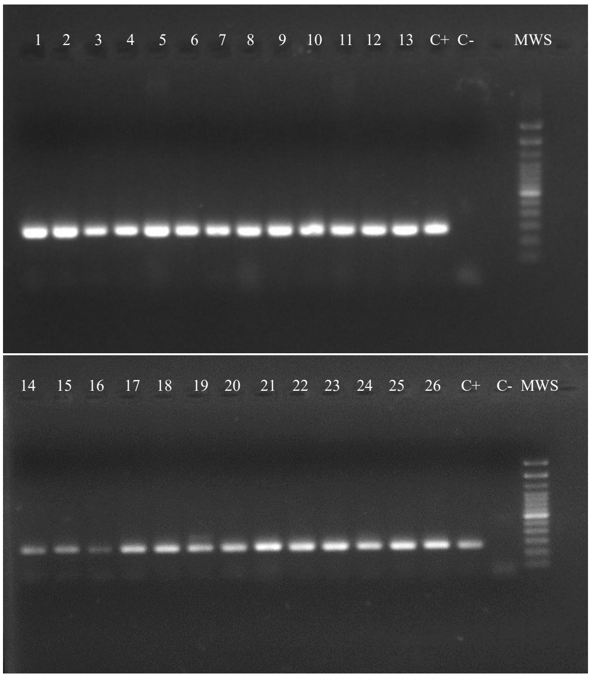

Supplementary Figure 2. Agarose gel of PCR products amplified using the specific primers for the detection of *PirAB* toxin genes (AHPND). PCR confirmation was conducted for 26 pools of stomachs (n = 13) and intestines (n = 13) of 65 shrimp with external signs of AHPND, used for microbiome analysis. Pools were obtained by combining five organs. Positive controls (C+) and negative controls (C-) were processed. MWS = Molecular Weight Standard (Gene Ruler 100 bp DNA Ladder). For the positive controls, an amplified product of 230 bp is observed.

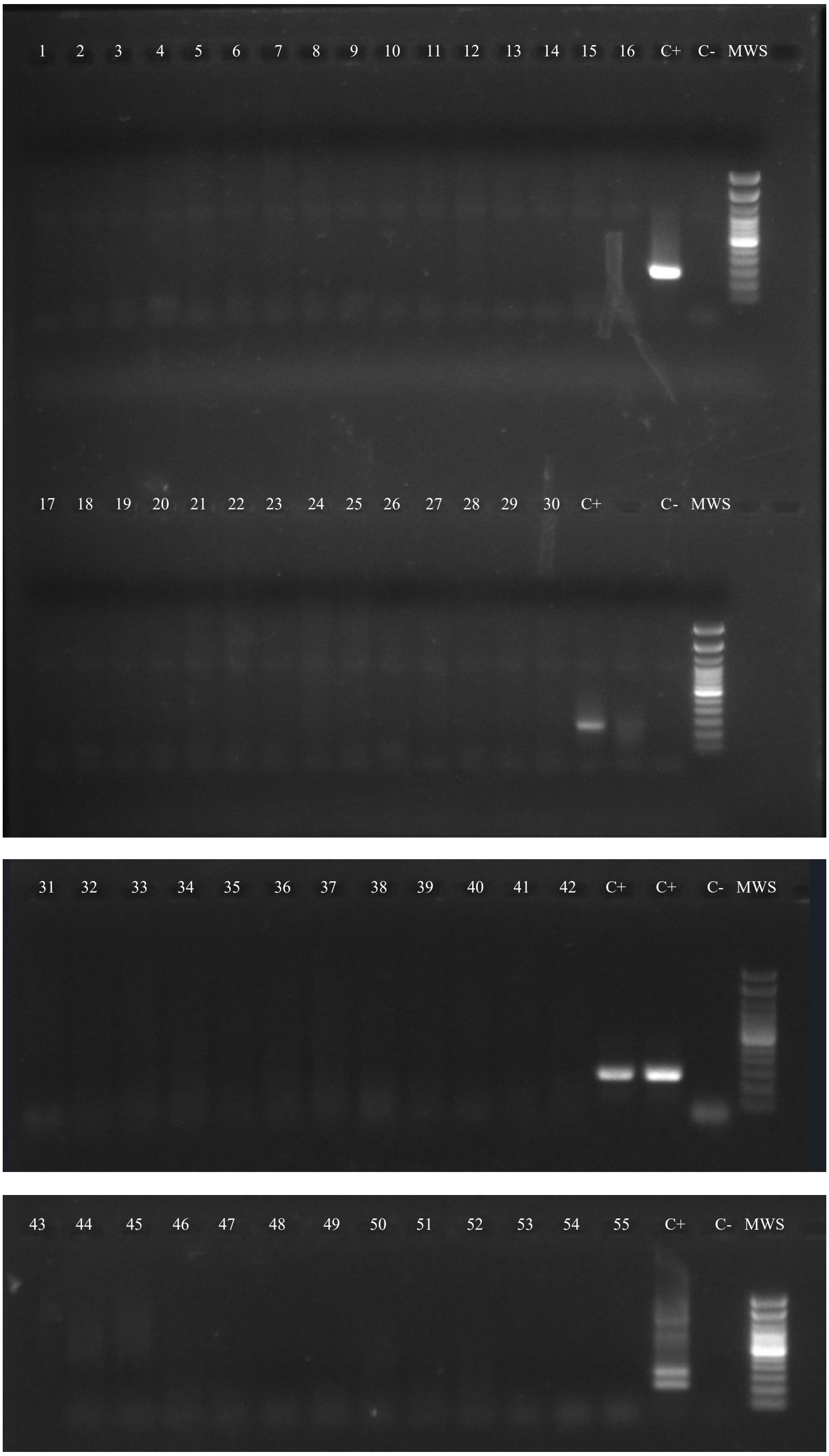

Supplementary Figure 3. Agarose gel of PCR products amplified using the specific primers for the detection of *PirAB* toxin genes (AHPND). PCR confirmation was conducted for the hepatopancreas of 55 apparently healthy shrimp, used for microbiome analysis. Positive controls (C+) and negative controls (C-) were processed. MWS = Molecular Weight Standard (Gene Ruler 100 bp DNA Ladder). For the positive controls, an amplified product of 230 bp is observed.

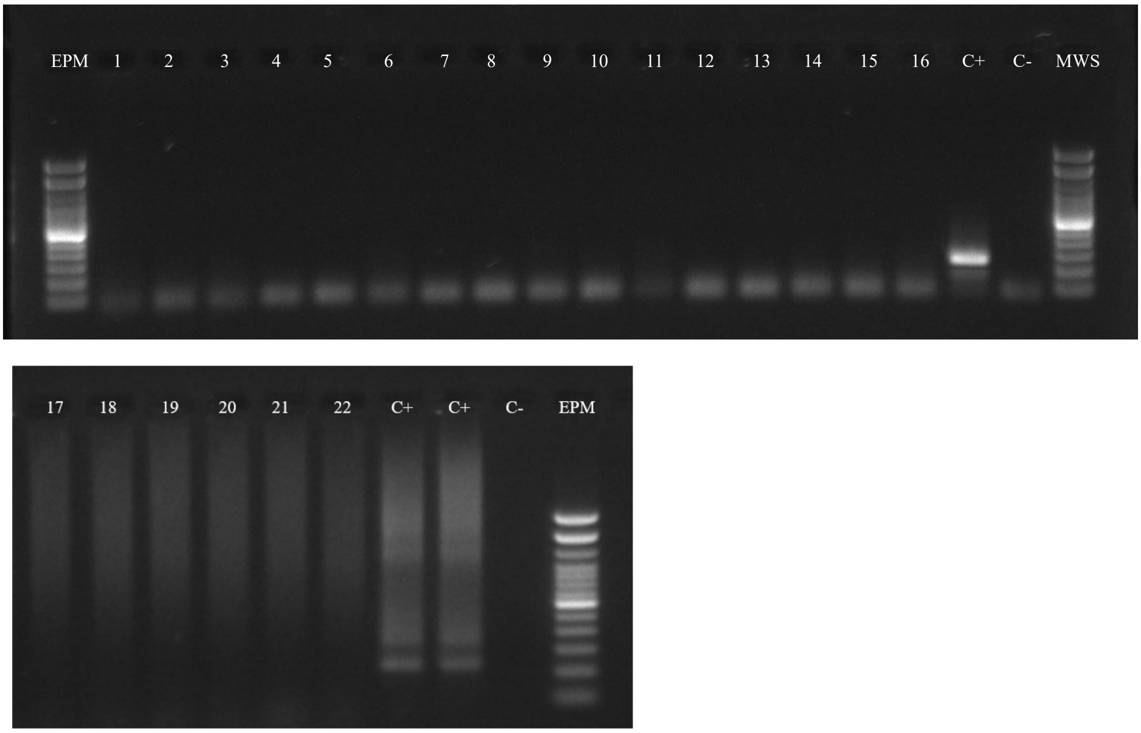

Supplementary Figure 4. Agarose gel of PCR products amplified using the specific primers for the detection of *PirAB* toxin genes (AHPND). PCR confirmation was conducted for 22 pools of stomachs (n=11) and intestines (n=11) of 55 apparently healthy shrimp, used for microbiome analysis. Pools were obtained by combining five organs. Positive controls (C+) and negative controls (C-) were processed. MWS = Molecular Weight Standard (Gene Ruler 100 bp DNA Ladder). For the positive controls, an amplified product of 230 bp is observed.

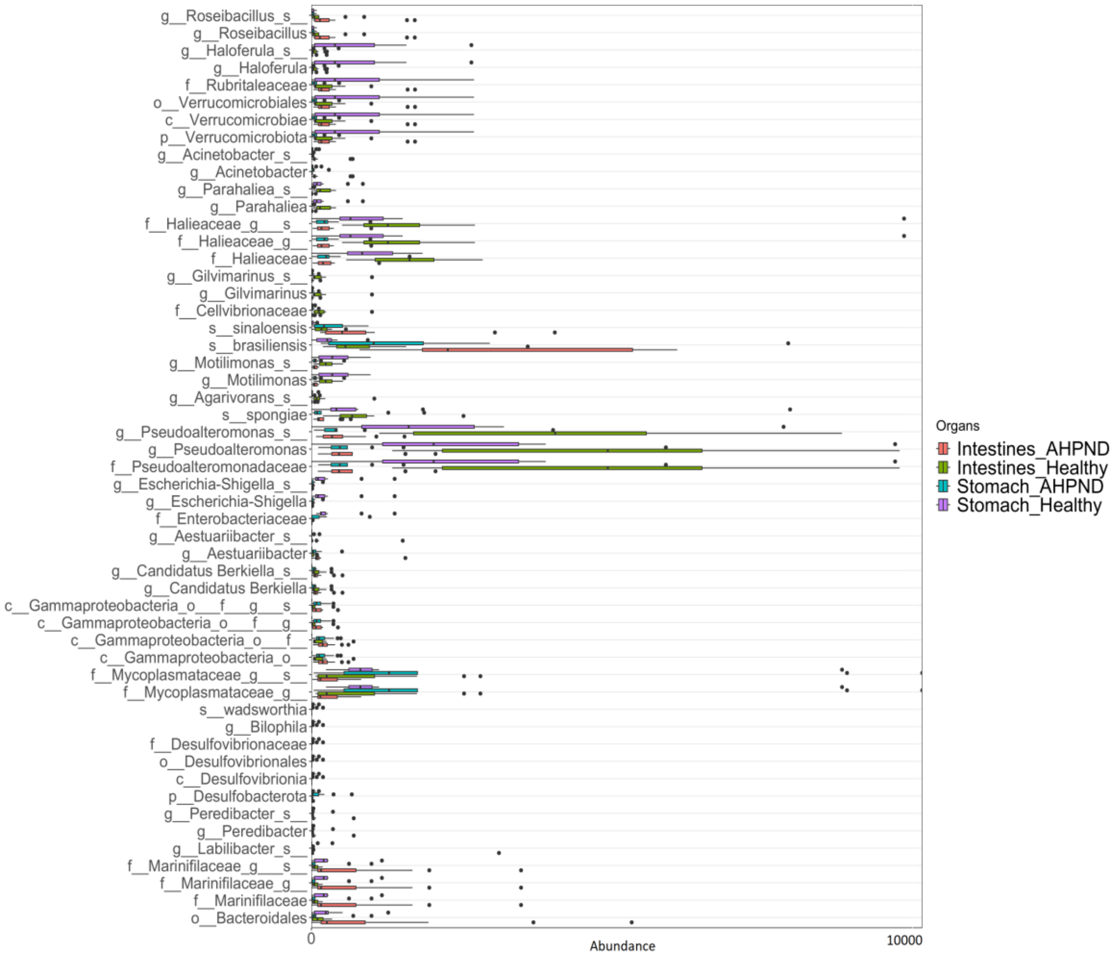

Supplementary Figure 5. Abundance of ASV signatures associated with stomachs and intestines of healthy and AHPND-affected *P. vannamei*.

Supplementary Table 1. Unique and common number of bacterial ASVs present in the stomachs and intestines of healthy and AHPND-affected *P. vannamei* shrimp.

	Health condition of shrimp	Organs	Number of ASVs	ASVs percentage (number of ASVs/4408)	Relative abundance (%)								
					Vibrio	***Catenococcus***	***Photobacterium***	***Agarivorans***	***Pseudomonas***	***Acinetobacter***	***Parabacteroides***	***Pseudoalteromonas***	***Noviherbaspirillum***
**Unique ASVs**	Healthy	Stomachs (11 pools)	1240	28.1	2.0	1.0	0.5	0.0	0.7	0.9	0.6	0.4	0.1
	AHPND	Stomachs (13 pools)	1015	23.0	2.0	1.2	1.0	0.2	1.1	1.1	0.3	0.4	0.4
	Healthy	Intestines (11 pools)	570	12.9	0.5	2.0	0.2	0.5	1.2	0.1	0.0	0.8	0.1
	AHPND	Intestines (13 pools)	519	11.8	4.0	4.0	1.7	0.0	3.0	0.5	0.0	0.1	0.3
**Common ASVs**	Healthy and AHPND	Stomachs (24 pools)	136	3.1	0.7	0.7	0.7	0.0	8.0	7.0	6.0	0.9	0.9
	Healthy and AHPND	Intestines (24 pools)	106	2.4	4.0	4.0	1.0	4.0	3.0	0.0	0.0	0.9	0.9
	Healthy	Stomachs and intestines (22 pools)	44	1.0	0.0	2.3	0.0	0.0	0.0	0.0	0.0	11.3	0.0
	AHPND	Stomachs and intestines (26 pools)	112	2.5	7.0	3.0	1.0	0.0	0.0	0.0	0.0	0.0	0.8
	Healthy and AHPND	Stomachs and intestines (48 pools)	234	5.3	14.0	6.0	3.0	2.1	2.0	0.4	0.0	4.0	2.0

Supplementary Table 2. Relative abundance of major bacterial taxa in the stomach and intestine of healthy and AHPND-affected *P. vannamei* juvenile shrimp.

**Health condition**	**Organs**	**Phylum**				**Order**				**Family**				**Genus**					
		Pseudomonadota	Bacillota	Bacteroidota	**Percentage (%)**	Vibrionales	Mycoplasmatales	Alteromonadales	**Percentage (%)**	Vibrionaceae	Mycoplasmataceae	Pseudoalteromonadaceae	**Percentage (%)**	*Photobacterium*	*Catenococcus*	*Pseudoalteromonas*	*Vibrio*	*Candidatus Bacilloplasma*	**Percentage (%)**
Healthy	Stomachs	64.3	27.1	5.3	96.7	37.5	22.0	15.7	75.2	37.5	22.0	11.2	70.8	16.2	13.4	11.2	6.6	12.1	59.5
	Intestines	70.5	21.1	6.7	98.3	37.8	19.2	21.1	78.1	37.8	19.2	14.4	71.4	5.9	18.6	14.4	13.2	17.2	69.3
Affected by AHPND	Stomachs	68.8	22.2	7.1	98.1	56.0	16.5	4.5	77.0	56.0	16.2	2.0	74.2	25.8	18.8	1.8	10.5	6.9	63.8
	Intestines	84.9	7.6	5.4	97.9	75.1	4.2	4.5	83.8	75.1	4.2	1.4	80.7	15.8	32.3	1.4	26.8	3.7	80.0
